# The miRNA-185-5p/STIM1 Axis Regulates the Invasiveness of Nasopharyngeal Carcinoma Cell Lines by Modulating EGFR Activation-Stimulated Switch from E- to N-Cadherin

**DOI:** 10.3390/molecules28020818

**Published:** 2023-01-13

**Authors:** Yue Luo, Jiaxiang Ye, Yayan Deng, Yujuan Huang, Xue Liu, Qian He, Yong Chen, Qiuyun Li, Yan Lin, Rong Liang, Yongqiang Li, Jiazhang Wei, Jinyan Zhang

**Affiliations:** 1 Department of Medical Oncology, Guangxi Medical University Cancer Hospital, Nanning 530021, China; 2 Institute of Biopharmaceutical and Health Engineering, Tsinghua Shenzhen International Graduate School, Tsinghua University, Shenzhen 518055, China; 3 Department of Otolaryngology & Head and Neck, The People’s Hospital of Guangxi Zhuang Autonomous Region, Nanning 530021, China; 4 Institute of Oncology, Guangxi Academy of Medical Sciences, Nanning 530021, China

**Keywords:** miRNA-185-5p, STIM1, epithelial-mesenchymal transition, tumor invasion, nasopharyngeal carcinoma

## Abstract

Distant metastasis remains the primary cause of treatment failure and suggests a poor prognosis in nasopharyngeal carcinoma (NPC). Epithelial-mesenchymal transition (EMT) is a critical cellular process for initiating a tumor invasion and remote metastasis. Our previous study showed that the blockage of the stromal interaction molecule 1 (STIM1)-mediated Ca^2+^ signaling blunts the Epstein–Barr virus (EBV)-promoted cell migration and inhibits the dissemination and lymphatic metastasis of NPC cells. However, the upstream signaling pathway that regulates the STIM1 expression remains unknown. In this follow-up study, we demonstrated that the miRNA-185-5p/STIM1 axis is implicated in the regulation of the metastatic potential of 5–8F cells, a highly invasive NPC cell line. We demonstrate that the knockdown of STIM1 attenuates the migration ability of 5–8F cells by inhibiting the epidermal growth factor receptor (EGFR) phosphorylation-induced switch from E- to N-cadherin in vitro. In addition, the STIM1 knockdown inhibited the locoregional lymphatic invasion of the 5–8F cells in mice. Furthermore, we identified miRNA-185-5p as an upstream regulator that negatively regulates the expression of STIM1. Our findings suggest that the miRNA-185-5p/STIM1 axis regulates the invasiveness of NPC cell lines by affecting the EGFR activation-modulated cell adhesiveness. The miRNA-185-5p/STIM1 axis may serve as a potentially effective therapeutic target for the treatment of NPC.

## 1. Introduction

Nasopharyngeal carcinoma (NPC) is a malignant disease that originally develops in the mucosal epithelium of the nasopharynx, with an unequal ethnic and geographical distribution, and is the most epidemic head and neck malignancy in southern China and Southeast Asia [1,2]. It has been well established that NPC pathogenesis closely correlates with an Epstein–Barr virus (EBV) latent infection, regional environmental factors, genetic susceptibility, and genomic instability [1,3]. Widely applied intensity-modulated radiation therapy (IMRT) has led to a local control rate of over 90% in non-metastatic NPC [4,5]. However, in a large proportion of patients with locally advanced NPC, this malignancy shows a strong tendency to involve regional lymph nodes and develop remote metastases. Distant metastasis strongly suggests an unfavorable clinical prognosis and remains the leading cause of tumor-associated death in NPC [1,4,6].

The epithelial-mesenchymal transition (EMT) is profoundly implicated in multiple malignant characteristics in human cancers, such as invasion, metastasis, and resistance to therapy [7,8]. Various signaling pathways are involved in manipulating the EMT program, such as the rapid transient phosphorylation of multiple downstream signaling proteins mediated by the activation of the epidermal growth factor receptor (EGFR) [9,10]. A store-operated Ca^2+^ entry (SOCE) controlled by the stromal interacting molecule 1 (STIM1) is a predominant mechanism for evoking a Ca^2+^ influx in non-excitatory cells, including NPC cells [11]. Our previous study showed that an EBV infection amplifies the EGF-induced Ca^2+^ responses by enhancing the intracellular STIM1 aggregation, thereby promoting EMT in NPC cells, yet the upstream signaling pathway that regulates the expression of STIM1 is still poorly understood [12].

The present study aimed to explore the role of STIM1 in the EGFR activation-mediated malignant behavior and the upstream regulatory molecule of STIM1 in NPC cell lines. Herein, we showed that the silencing of STIM1 resulted in the inhibitions of cell migration, invasion, and metastasis in 5–8F cells, a highly invasive NPC cell line. In addition, by querying the miRBase and miRDB biological databases, miRNA-185-5p was identified as a potential upstream regulatory molecule of STIM1. Thus, we further verified the regulatory relationship between miRNA-185-5p and STIM1 and demonstrated that miRNA-185-5p negatively regulates the STIM1 mRNA expression by binding to its 3′ untranslated region (3′ UTR).

## 2. Results

### 2.1. Knockdown of STIM1 Inhibits EGF-Induced Migration of 5–8F Cells

**Figure 1:**
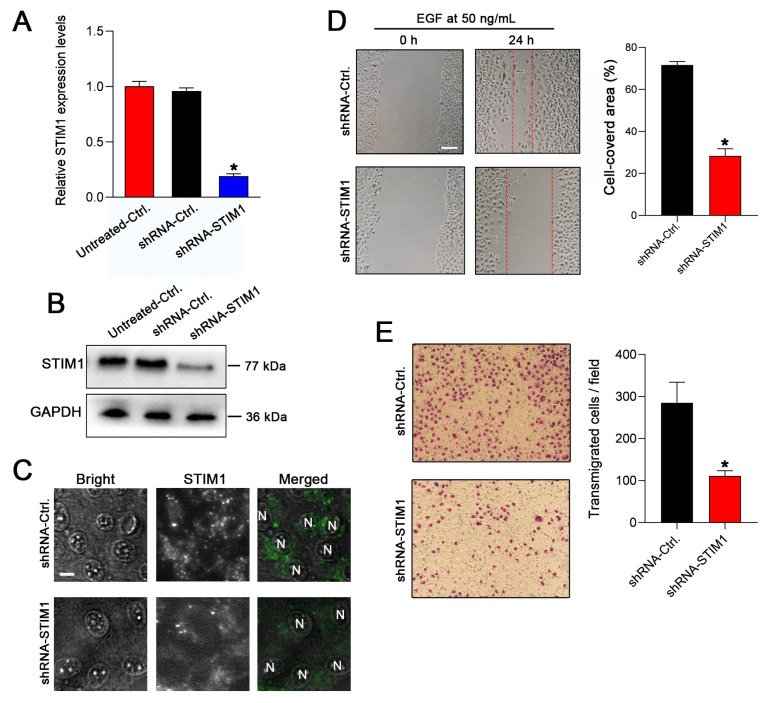
Knockdown of STIM1 inhibits the EGF-stimulated migration of 5–8F cells. (**A**) The expression levels of STIM1 in 5–8F cells transduced with shRNA-Ctrl (nonsense) or shRNA-STIM1 vector were determined by RT-qPCR. (**B**) The expression levels of STIM1 in the shRNA-Ctrl or shRNA-STIM1 5–8F cells were detected by Western blotting. (**C**) The intracellular STIM1 aggregation was analyzed by immunofluorescence staining upon EGF stimulation. Bar = 10 μm. (**D**) Wound-healing assay was performed to evaluate the effect of STIM1 knockdown on EGF-stimulated cell migration. Representative photographs of migratory cells were captured after incubation of cells with extracellular EGF for 24 h. Bar = 100 μm. Cell migration was quantified by calculating the cell-covered area (right) (n = 5 per group). (**E**) The transwell assay was performed to evaluate cell migration. Representative photographs of the membrane-penetrating cells stained with Giemsa from each group are shown. The average numbers of membrane-penetrating cells from a random field are shown (right panel) (n = 6 per group). Data are presented as mean ± SD of three independent experiments. (* *p* < 0.05, Student’s *t*-test).

Both highly invasive 5–8F cells and non-invasive 6–10B cells were derived from their parental NPC cell line, SUNE1 [13]. In our earlier studies, we showed that EGF-induced Ca^2+^ signaling was significantly enhanced in 5–8F cells compared with the signaling in 6–10B cells, suggesting that a Ca^2+^ signal amplification is related to the invasiveness of NPC cell lines [12]. Therefore, 5–8F cells were used to explore the pathway through which STIM1 modulates the invasiveness of the NPC cell lines as well as the upstream pathway that potentially regulates STIM1. 5–8F cells were stably transfected with the lentiviral vector carrying shRNA-STIM1 or shRNA-Ctrl, and the transfection efficiency was elucidated by calculating the percentages of the green fluorescence (GFP)-positive cells (Supplementary Figure S1). The decreased expression of STIM1 was confirmed by RT-qPCR and Western blot analysis (Figure 1A,B). The reduced STIM1 expression was also detected in the immunofluorescent analysis of an intracellular STIM1 aggregation in the shRNA-STIM1 cells (Figure 1C). In our previous study, we showed that STIM1 is involved in modulating the migratory ability of EBV-positive cells [12]. To clarify whether STIM1 also regulates the migration capacity of EBV-negative NPC cell lines, the migration of 5–8F cells was determined using a wound healing assay upon an EGF stimulation. The results showed that the migration rate of shRNA-STIM1 cells was significantly lower than that of shRNA-Ctrl cells (Figure 1D). Transwell assays were performed to evaluate the effect of the knockdown of STIM1 on the migration of 5–8F cells, and it was found that the number of cells crossing the filter membrane in the shRNA-STIM1 group was significantly reduced than that in the shRNA-Ctrl group (Figure 1E). These results indicate that STIM1 knockdown significantly inhibited the EGF-stimulated migration of 5–8F cells.

### 2.2. STIM1 Regulates EGFR Activation-Stimulated EMT

**Figure 2:**
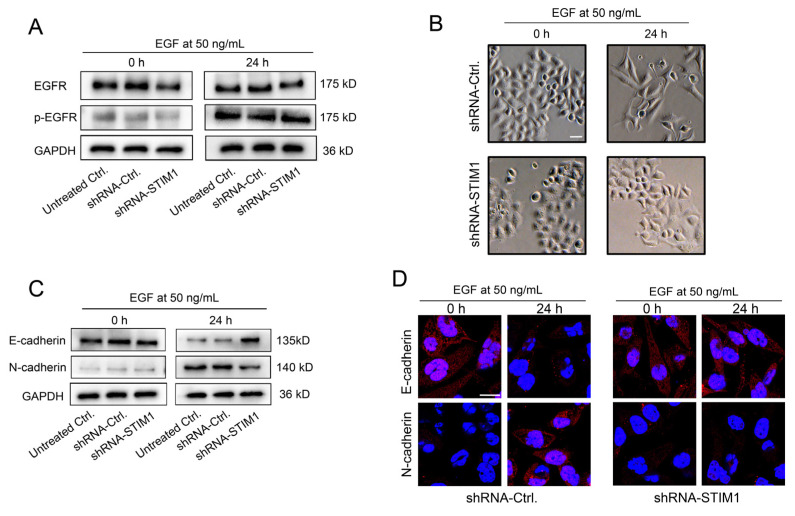
Knockdown of STIM1 inhibits the EGFR activation-stimulated switch from E- to N-cadherin. (**A**) The effect of STIM1 knockdown on EGFR expression and phosphorylation was evaluated using Western blotting. The shRNA-untreated cells served as blank controls. (**B**) Morphological changes in 5–8F cells transfected with lentiviral vectors carrying shRNA-Ctrl or shRNA-STIM1 before and after EGF stimulation. Bar = 20 μm. (**C**) The effect of STIM1 knockdown on the EGF-stimulated switch from E-cadherin to N-cadherin was examined using Western blotting. (**D**) Changes in the expression of E-cadherin and N-cadherin were determined using cellular immunofluorescence. Bar = 10 μm.

We previously reported that an EBV infection amplified the EGF-evoked Ca^2+^ responses by enhancing STIM1 aggregation [12]. In this follow-up study, we further explored the role of STIM1 in the EGFR activation-stimulated switch from E- to N-cadherin in 5–8F cells. We first verified by Western blotting that the STIM1 knockdown did not directly affect the total expression or phosphorylation of the EGFR (Figure 2A). Upon an extracellular EGF stimulation, some 5–8F cells transformed from epithelioid to fusiform mesenchymal morphology in the shRNA-Ctrl group. However, EGF-induced morphological changes were less obvious in STIM1-depleted cells (Figure 2B). EMT refers to a cellular biological program by which epithelial cells acquire the mesenchymal phenotypes required for migration and invasion, which is defined by the loss of epithelial signature molecules and the upregulation of mesenchymal markers [7]. Using Western blotting and cell immunofluorescence staining, it was found that the expression of E-cadherin was decreased while N-cadherin was increased in shRNA-Ctrl cells, and the switch from E-cadherin to N-cadherin was blocked by the silencing of STIM1 (Figure 2C,D). These results suggest that STIM1 modulates the invasiveness of NPC cell lines by controlling the EGFR phosphorylation-modulated cell adhesiveness.

### 2.3. Knockdown of STIM1 Impairs Locoregional Lymphatic Metastasis

**Figure 3:**
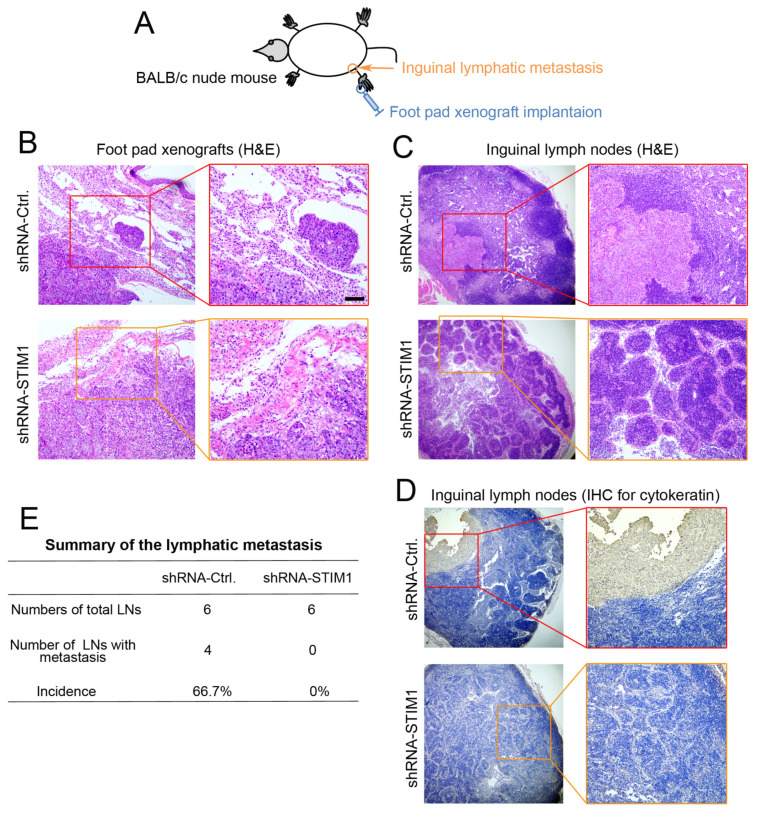
Knockdown of STIM1 reduces the regional lymphatic metastasis in vivo. (**A**) Schematic diagram illustrates an inguinal lymph node metastasis model of mice. (**B**) Representative photographs of H&E-stained xenograft tumors in the foot pad of shRNA-Ctrl or shRNA-STIM1 groups. The tumor emboli were observed in the surrounding vasculature in the shRNA-Ctrl group (red box). The shRNA-STIM1 group had no tumor emboli in the surrounding vasculature. Bar = 100 μm. (**C**) H&E staining of lymph node sections from the two groups. Local-regional metastases were found in the inguinal lymph node from the shRNA-Ctrl group (red box) but none in those from the shRNA-STIM1 group. (**D**) Immunohistochemical analysis for cytokeratin was performed to confirm the epithelial origin of lymph node metastasis (red box). (**E**) Summary of the incidence of lymph node metastasis in each group (*p* < 0.05, one-sided Fisher’s exact test).

To determine whether STIM1 regulates the lymphatic metastasis of 5–8F cells, we employed a mouse model for evaluating inguinal lymph node metastasis in vivo (Figure 3A), as described previously [12]. Forty days post-implantation, the primary foot pad xenografts and ipsilateral inguinal lymph nodes were isolated and collected. H&E staining of the sections showed that the xenograft tumors in the shRNA-Ctrl group grew invasively and tumor emboli were formed in the nearby vasculature, exhibiting a highly aggressive property. In contrast, shRNA-STIM1 xenograft tumors grew in a non-invasive manner, and no tumor emboli were formed (Figure 3B). In addition, locoregional lymphatic metastases were observed in the ipsilateral lymph nodes in the shRNA-Ctrl group. Lymph-infiltrating tumor cells were recognized to have large nuclear deformities and obvious atypia. In comparison, the shRNA-STIM1 group showed the integrity of lymph nodes without the legible infiltration of tumor metastases (Figure 3C). Furthermore, immunohistochemical analysis showed that lymphatic metastases in the shRNA-Ctrl group were positive for pan-cytokeratin, a classic epithelial marker, which confirmed the epithelial origin of the metastases formed by the 5–8F xenografts, whereas there was no positive staining in the shRNA-STIM1 group (Figure 3D). In summary, 66.7% (4/6) of mice in the shRNA-Ctrl group developed inguinal lymph node metastasis, whereas 0% (0/6) in the shRNA-STIM1 group developed lymph node metastasis (Figure 3E). These results indicate that the knockdown of STIM1 suppressed the lymphatic metastatic potential of invasive 5–8F cells.

#### 2.4. miRNA-185-5p Negatively Regulates STIM1 Expression

**Figure 4:**
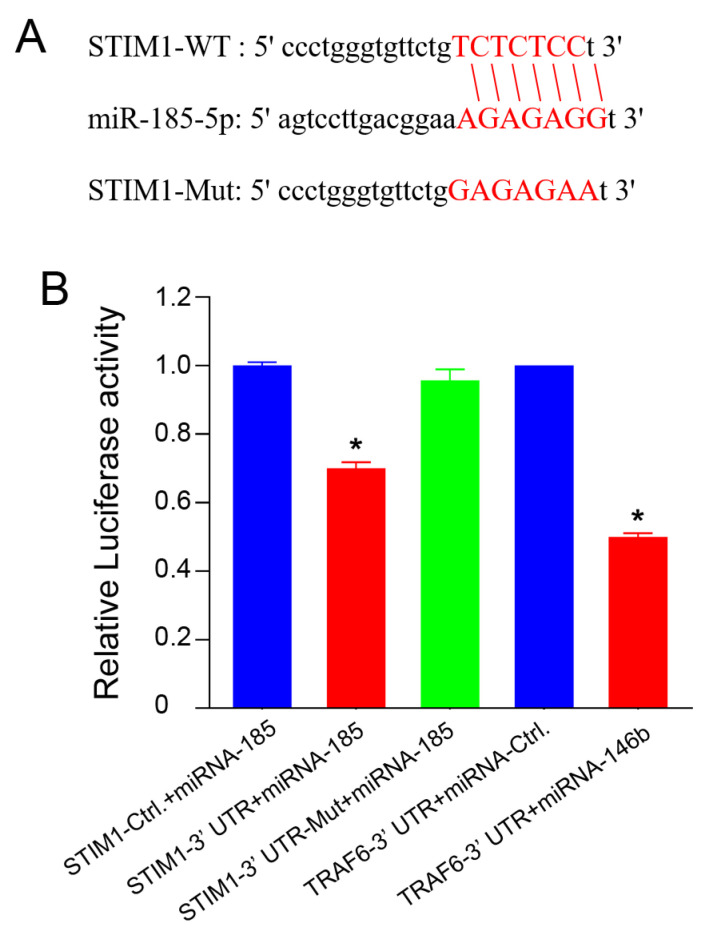
STIM1 is a direct target gene of miRNA-185-5p. (**A**) miRNA-185-5p can bind to the wild-type 3′ UTR of STIM1 but not to the mutant 3′ UTR of STIM1. The involved sequences are highlighted in red. (**B**) miRNA-185-5p significantly suppressed the luciferase activity of wild-type 3′ UTR of STIM1 but did not affect that of the mutant 3′ UTR of STIM1. The positive control was used to evaluate the effectiveness of the entire transfection detection system. Data are expressed as mean ± SD (* *p* < 0.05, Student’s *t*-test).

To investigate the upstream regulatory mechanism of the STIM1s expression, we queried miRBase and miRDB biological databases and found that miRNA-185-5p is a potential upstream regulatory molecule of STIM1 (Figure 4A). After introducing a mutation in the miRNA-185-5p binding site of the 3′ UTR of STIM1 mRNA, the dual-luciferase reporter gene assay indicated that the transcription of the wild-type STIM1 gene was inhibited, whereas that of the gene with the mutation site was not affected, indicating that miRNA-185-5p bound to the 3′ UTR of STIM1 and inhibited the expression of STIM1 (Figure 4B).

**Figure 5:**
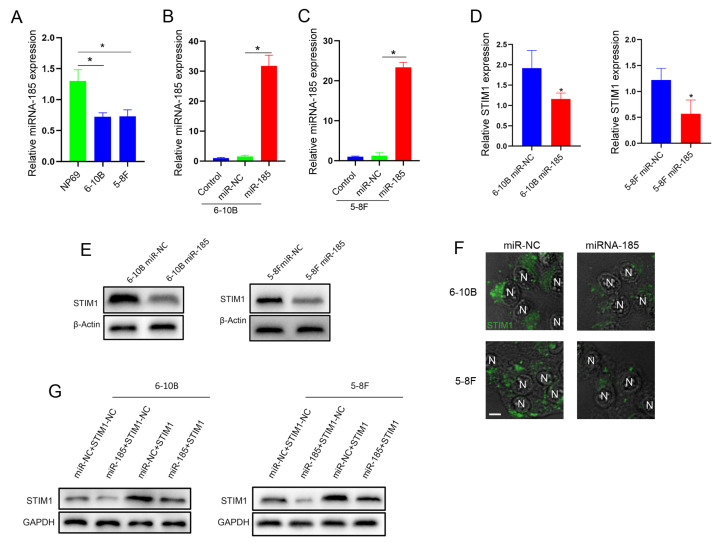
Targeted regulation of STIM1 expression by miRNA-185-5p. (**A**) RT-qPCR was performed to determine the relative expression of miRNA-185 in a nasopharyngeal epithelial cell line NP69, and the two NPC cell lines 6–10B and 5–8F. (**B**) Quantitative analysis of miRNA-185 levels in the indicated groups assessed by RT-qPCR. (**C**) Quantitative analysis of miRNA-185 levels in the indicated groups assessed by RT-qPCR. (**D**) Quantitative analysis of STIM1 levels in 6–10B or 5–8F cells overexpressing miRNA-185 assessed by RT-qPCR. (**E**) Expression of STIM1 in 6–10B or 5–8F cells overexpressing miRNA-185 was assessed using Western blotting. (**F**) Aggregation of intracellular STIM1 was detected in 6–10B or 5–8F cells overexpressing miRNA-185 with immunofluorescence staining. Bar = 10 μm. (**G**) The rescue experiment was carried out to confirm the targeted regulation of STIM1 by miRNA-185 in 6–10B or 5–8F cells. The levels of proteins were assessed using Western blotting. Data are expressed as mean ± SD of three independent experiments. (* *p* < 0.05, Student’s *t*-test).

We next examined the expression of miRNA-185-5p by RT-qPCR in the normal nasopharyngeal epithelial cell line NP69 and the NPC cell lines 6–10B and 5–8F. Our results showed that the miRNA-185-5p expression in 6–10B or 5–8F cells was lower than that in NP69 cells (Figure 5A). To further verify the targeted regulation of STIM1 by miRNA-185-5p, we constructed an NPC cell model in which the miRNA-185-5p was overexpressed (Figure 5B,C). The introduction of miRNA-185-5p into 6–10B and 5–8F cells reduced the STIM1s expression (Figure 5D,E). In addition, the decreased STIM1s expression was further confirmed by the immunofluorescence staining of cytosolic STIM1 aggregation (Figure 5F). In subsequent rescue experiments, the overexpression of STIM1 successfully reversed the reduction in the STIM1 expression in miRNA-185-5p-overexpressing cells (Figure 5G). Thus, miRNA-185-5p targets and negatively regulates the STIM1 expression in invasive 5–8F cells.

## 3. Discussion

The concentration of Ca^2+^ in the cytoplasm is strictly controlled within a specified range to accurately transfer extracellular signals into the cytoplasm [14]. SOCE is a dominant pathway in the generation of calcium signals in non-excitable cells and maintains cytosolic Ca^2+^ homeostasis, together with ATP-Ca^2+^ pumps on the endoplasmic reticulum (ER) membrane and plasma membrane [15,16]. The dysregulation of SOCE-mediated Ca^2+^ signaling is implicated in various malignant phenotypes of human cancer cells, including proliferation, invasion, metastasis, immune escape, tumor-associated angiogenesis, EMT, and drug resistance [17,18,19,20,21,22]. STIM1, an intracellular Ca^2+^ sensor, serves as a key component of SOCE. The depletion of ER Ca^2+^ induces STIM1 to accumulate intracellularly, which is then further transported to the ER-plasma membrane junction, where it directly binds and activates the opening of ORAI1 channels, subsequently triggering a Ca^2+^ influx [23,24]. STIM1 is upregulated in various human malignancies, including liver, breast, cervical, and colon cancers [19,24,25,26]. We previously demonstrated that STIM1 is actively expressed in primary NPC tissues and associated with the degree of regional lymph node metastasis (N stage) [12]. Abnormally expressed STIM1 disrupts the dynamic balance of intracellular Ca^2+^ by enhancing the SOCE activity, thus triggering the malignant transformation of tumor cells, such as the switch from E- to N-cadherin shown in our study.

Metastasis is a primary obstacle in cancer treatment and a leading cause of death in most cancers, including NPC. Cell migration is considered to be an initializing step for tumor metastasis and progression, enabling cancer cells to escape from their primary sites and disseminate through circulation [27]. SOCE is co-regulated by ORAI1 and STIM1 and is strictly required for breast cancer cell migration. Blocking SOCE can reduce the focal adhesion turnover, thereby enhancing cell adhesion and weakening migration ability [28]. Chen et al. showed that expression of STIM1 is crucial for the EGF-driven cell migration in cervical cancer [19]. In our earlier study, we found that among the various Ca^2+^ channels, only a Ca^2+^ influx through SOCE regulates an NPC cell migration [29]. In this follow-up study, we further showed that the reduction in STIM1 inhibited the EGF-induced migration of 5–8F cells. In addition, we confirmed that the reduction in STIM1 could inhibit a local invasion and lymphatic metastasis of 5–8F cells in mice. Furthermore, STIM1 affected the migration capability by regulating the EGF-induced switch from E-cadherin to N-cadherin without affecting the EGFR phosphorylation in 5–8F cells. The transition from the epithelial to mesenchymal state allows cancer cells to acquire an aggressive phenotype that is required for a metastatic progression, particularly to escape from the primary site and spread into the circulatory bloodstream [30,31]. The essential role of STIM1-mediated Ca^2+^ signaling in EMT induction has also been reported. The ectopic expression of STIM1 is critical for the acquisition of mesenchymal features in colorectal cancer cells [26]. The high expression level of STIM1 correlates with an unfavorable prognosis in colorectal cancer [26]. Xia et al. indicated that the knockdown of STIM1 inhibits the proliferation, migration, invasion, and EMT of gastric cancer cells [20]. Zhang et al. showed that STIM1 promotes the EMT induced by TGF-β by modulating SOCE in breast cancer cells [32]. Taken together, STIM1-mediated Ca^2+^ signaling actively participates in the regulation of the tumor’s invasiveness by modulating the cell adhesiveness.

The aberrantly enhanced expression of the EGFR or its cognate ligands leads to the overactivation of the EGFR signaling pathway, which drives the development and progression of human cancers [33,34,35,36]. In this study, we showed that the knockdown of STIM1 inhibits the EGFR activation-induced switch from E-cadherin to N-cadherin in an NPC cell line, 5–8F. To verify the effect of STIM1 on the EGFR signaling pathway, we first examined the expression levels of the EGFR and phosphorylated EGFR in shRNA-Ctrl and shRNA-STIM1 cells. Our results indicated that the knockdown of STIM1 did not affect the EGFR or its phosphorylation following the treatment with EGF, suggesting that STIM1 manipulates the EGFR activation-modulated cell adhesiveness by regulating the EGFR activation-evoked SOCE without directly interfering with the EGFRs activation. Indeed, the STIM1-mediated SOCE activation promotes migration and invasion in hepatocellular carcinoma cells by modulating the focal adhesion turnover [25]. In addition, the blockage of STIM1-mediated Ca^2+^ signaling enhances cell adhesion by inhibiting the turnover of focal adhesions, which hampers the rapid migration of cells, including metastatic cancer cells [28].

MicroRNAs (miRNAs) are a class of endogenous non-coding small RNA molecules that downregulate the target gene expression level by binding to the 3′-UTR of mRNAs [37,38]. miRNAs actively contribute to various tumor biological behaviors, including cell proliferation, apoptosis, EMT, local invasion, remote metastasis, and tumor angiogenesis [39,40,41,42,43,44]. miRNA-185 regulates the functional characteristics that are responsible for angiogenesis in human microvascular endothelial cells by targeting STIM1 [45]. Moreover, miRNA-185 modulates the metastatic properties of colon cancer cells by targeting STIM1 [26]. However, whether miRNA-185 affects the malignant properties of NPC cells by regulating the STIM1 expression remains unclear. In this study, a dual-luciferase reporter gene system and rescue experiments were employed to testify that miRNA-185-5p binds to the 3′ UTR of STIM1 and inhibits its expression in NPC cell lines. Since a single miRNA can simultaneously regulate multiple downstream target genes, the intervention of miRNA may have a better therapeutic value than that of protein-encoding genes [46].

In our previous study, blocking STIM-dependent Ca^2+^ signaling in NPC cells attenuated the EMT program, thereby blunting the EBV-promoted invasiveness [12]. In addition, we previously demonstrated that the STIM1-dependent Ca^2+^ signaling pathway is implicated in EBV-driven tumor angiogenesis in NPC [22]. In the present study, we further demonstrated that the depletion of STIM1 inhibited cell migration and lymphatic metastasis in the highly invasive NPC cell line 5–8F. Furthermore, miRNA-185-5p was identified for the first time as an upstream regulator of STIM1 in NPC cell lines. However, the following unsolved issues still need to be addressed in the future: (1) the effect of the expression of miRNA-185 on the invasiveness of NPC cells should be verified, (2) the role of miRNA-185 in the regulation of the STIM1 expression still needs to be further testified in NPC tissue samples, (3) the correlation between the expression levels of the STIM1 and EMT makers such as E- and N-cadherin should be further analyzed in primary tumor tissues, and (4) the clinical significance of the expression levels of miRNA-185 for predicting prognosis is yet to be evaluated by a well-designed follow-up study.

## 4. Materials and Methods

### 4.1. Cell Culture

The highly metastatic (5–8F) and non-metastatic (6-10B) NPC cell lines are the subpopulations that originated from SUNE1 cells [13,47]. The cells were cultured in the Roswell Park Memorial Institute (RPMI) 1640 medium supplemented with 5% fetal bovine serum (FBS), 100 μg/mL of streptomycin, and 100 U/mL of penicillin (Thermo Fisher Scientific, Waltham, MA, USA). NP69 cells, a nasopharyngeal epithelial cell line, were cultured in a keratinocyte serum-free medium (Thermo Fisher Scientific) supplemented with antibiotics as described above. The NP69 cell line was kindly provided by Professor Sai-Wah Tsao (the University of Hong Kong, Hong Kong, China). The cells were maintained in an incubator at 37 °C under 5% CO_2_. The cell lines used in the present study were verified by short tandem repeat (STR) analysis to exclude the potential contamination between cells.

### 4.2. Knockdown of STIM1

The lentiviral vector GV248, carrying a short hairpin RNA against STIM1 (shRNA-STIM1) or negative control short hairpin RNA (shRNA-Ctrl), was used in this study (GeneChem Technology Co. Ltd., Shanghai, China). The shRNA sequence against STIM1 was GGGAAGACCTCAATTACCA, and the nonsense sequence TTCTCCGAACGTGTCACGT was used as the scrambled shRNA control. The 5–8F cells were transfected with the lentivirus vector according to the manufacturer’s instructions, and puromycin (1 µg/mL, Thermo Fisher Scientific) was used to screen the cell clones for a stable infection. The sequence encoding green fluorescence (GFP) was simultaneously introduced into the NPC cell line 5–8F. The percentage of GFP-expressing cells was determined to evaluate the transfection efficiency. A real-time quantitative polymerase chain reaction (RT-qPCR) was applied to detect the relative expression levels of STIM1, and the primer sequences and product lengths are shown in Supplementary Table S1. The expression of STIM1 was also detected by Western blotting using an antibody against STIM1 (1:1000, cat. No. ab57834, Abcam, Cambridge, UK). The GAPDH expression was used as the internal reference (1:1000; cat. no. ab8245, Abcam).

### 4.3. Wound Healing Assay

Briefly, 6 × 10^5^ cells were collected from each group (shRNA-STIM1 or shRNA-Ctrl) and seeded into the wells and were then incubated in the serum-free medium for 24 h until reaching an approximately 90% confluence. The monolayer of cells growing in 6-well plates was scratched with a 200 μL sterile pipette tip to generate a wound. The wells were rinsed with phosphate-buffered saline (PBS) three times, and the serum-free medium supplemented with a human recombinant epidermal growth factor (EGF; 50 ng/mL, Invitrogen) was applied, followed by incubation at 37 °C for 24 h. Five random fields were selected and photographed after 24 h, and the capacity of the cell migration was quantified by calculating the cell-covered area (%) utilizing a quantitative wound healing image analysis software (Ibidi, Martinsried, Germany).

### 4.4. Transwell Assay

Cell migration assays were performed in a 24-well transwell chamber (Corning Inc., Corning, NY, USA). A total of 1 × 10^5^ cells (shRNA-STIM1 or shRNA-Ctrl) were suspended in a 200 μL serum-free medium and were added to the upper chamber (pore size of the membrane = 8 µm). The serum-free medium containing EGF (50 ng/mL) was added to the lower chamber as described in our previous study [12]. The cells migrate from the upper to the lower chambers under the chemotaxis effect of the EGF. Following incubation for 24 h at 37 °C, membrane-penetrating cells were stained with Giemsa and counted under an inverted microscope (Leica Microsystems GmbH, Tokyo, Japan).

### 4.5. Western-Blot and Cell Immunofluorescence Staining

To study the role of STIM1 in the EGFR activation-induced signaling pathway, 5–8F cells were stimulated with extracellular EGF (50 ng/mL). The total protein was isolated following the lysis of cells in RIPA buffer containing a protease inhibitor cocktail (Thermo Fisher Scientific). To detect the expression of the EGFR, phosphorylated EGFR (pEGFR), and the EMT markers, including E-cadherin and N-cadherin, Western blotting was performed using antibodies against the EGFR [1:1000, cat. no. 4267S, Cell Signaling Technology (CST), [Boston, MA, USA], pEGFR (1:1000, cat. no. 3777S, CST), E-cadherin (1:1000; Cat. no. ab76055; Abcam), and N-cadherin (1:1000; cat. no. ab18203, Abcam). The cytosolic aggregation of STIM1 upon an extracellular EGF stimulation was evaluated by immunofluorescence staining using an anti-STIM1 antibody (1:200; Cat. no. ab62031; Abcam) and Alexa Fluor 647 goat anti-rabbit lgG (4 μg/mL; Invitrogen), as described in our previous study [12]. The expression of E-cadherin and N-cadherin was also detected by immunofluorescence staining. The cells were fixed in 4% paraformaldehyde and permeabilized with PBS containing 0.5% Triton X-100. After blocking with 2% bovine serum albumin (BSA) for 30 min, anti-E-cadherin (1:200, cat. no. ab76055, Abcam) and anti-N-cadherin antibodies (1:200, cat. no. ab18203, Abcam) were added for a co-incubation and were stained with a Cy3-labeled fluorescent secondary antibody (1:500, Biyuntian Biological Technology Co., LTD, Shanghai, China). The nuclei were counter-stained with DAPI (Biyuntian). The dishes were observed under a confocal laser scanning microscope (Olympus FV1000, Tokyo, Japan).

### 4.6. Inguinal Lymph Node Metastasis Model of Nude Mice

Female BALB/c nude mice (aged 4–6 weeks) were provided by the Animal Center of Guangxi Medical University and raised in the SPF animal laboratory. All the experimental procedures were approved by the Institutional Animal Care and Use Committee of the Guangxi Medical University Animal Center. Mice were randomly divided into the shRNA-STIM1 and shRNA-Ctrl groups (n = 6 per group). 5–8F cells (shRNA-STIM1 or shRNA-Ctrl) were injected into the muscle layer of the footpads of the mice to establish a model of inguinal lymph node metastasis, as previously described [48,49]. After 40 days of growth, the footpad xenografts and inguinal lymph nodes were isolated, paraffin-embedded, and cut into 5 μm tissue sections for routine hematoxylin and eosin (H&E) staining and immunohistochemical (IHC) analysis.

### 4.7. Dual-Luciferase Reporter Gene Assay

To verify the targeting relationship between STIM1 and miRNA-185-5p, 293T cells (1 × 10^5^) were cultured in 24-well plates and co-transfected with miRNA-185-5p mimics or negative control (NC), the renilla luciferase reporter, and luciferase reporter containing the wild-type or mutant 3′ UTR of STIM1. Transfections were repeated in three independent experiments. Forty-eight hours after transfection, a Dual-Luciferase Reporter Assay System (E1910; Promega, Madison, WI, USA) was employed to elucidate the luciferase activities. The combination of TRAF6-3′ UTR and miRNA-146b was used as a positive control to indicate the effectiveness of the entire transfection detection system.

### 4.8. Construction of NPC Cell Lines Overexpressing miRNA-185 or Overexpressing Both miRNA-185 and STIM1

The 6–10B and 5–8F cells in the logarithmic growth phase were transfected with the lentivirus carrying short hairpin RNA. The precursor of miRNA-185 was inserted into the Ubi-MCS-SV40-Cherry-IRES-neomycin vector (Genechem Co., CV622, Shanghai, China) to upregulate the expression of miRNA-185. STIM1 was inserted into the Ubi-MCS-3FLAG-CBh-gcGFP-IRES-puromycin vector (Genechem Co., GV492, Shanghai, China) to overexpress STIM1. The nonsense vector served as a negative control (NC).

### 4.9. Statistical Analysis

The experimental data were statistically analyzed using SPSS software (version 22.0; SPSS Inc., Chicago, IL, USA), and the normally distributed data are expressed as the mean ± standard deviation. The comparison of the means between the two groups was performed using Student’s *t*-test. Fisher’s exact test was used for the statistical analysis of the incidence of lymph node metastasis in mice. Differences were considered statistically significant when *p* < 0.05.

## 5. Conclusions

In the present study, it was established that miRNA-185-5p regulates the invasiveness of NPC cell lines by manipulating the expression of STIM1, which modulates the EGFR activation-induced switch from E- to N-cadherin. The miRNA-185-5p/STIM1 axis that controls cell adhesiveness could be an effective target for anti-metastatic therapy for treating NPC.

## Data Availability

Not applicable.

## Supplementary Materials

The following supporting information can be downloaded at: https://www.mdpi.com/article/10.3390/molecules28020818/s1, Figure S1: Transfection efficiency assay of shRNA-Ctrl or shRNA-STIM1; Figure S2: Lentiviral vector-mediated transfection of miRNA-185 or miRNA-control; Table S1: Primer sequence and product length.

## Sample Availability

Not available.
