# Supplementary material for: The miRNA-185-5p/STIM1 Axis Regulates the Invasiveness of Nasopharyngeal Carcinoma Cell Lines by Modulating EGFR Activation-Stimulated Switch from E- to N-Cadherin
Source: Molecules. 2023 Jan 13;28(2):818. doi: 10.3390/molecules28020818 (PMC9864293; doi:10.3390/molecules28020818)
Supplement: Supplementary file 1 [file molecules-28-00818-s001.zip › Supplementary Figure legend.pdf]

Supplementary Figure Legend

Figure S1. Transfection efficiency assay of shRNA-Ctrl or shRNA-STIM1. The green fluorescence (GFP) encoding sequence was transfected into 6-10B or 5-8F cells, which served as a fluorescent indicator for the successful transfection into cells. Bar =100 μm. Transfection efficiency was evaluated by calculating the percentage of GFP-positive cells (right panel). Data are expressed as mean ± SD of three random fields.

Figure S2. Transfection of miRNA-185 or miRNA-Ctrl. The red fluorescence encoding sequence was introduced into 6-10B or 5-8F cells, which served as a visible indicator for the successful transfection into cells. Bar =100 μm.
