# Supplementary material for: The miRNA-185-5p/STIM1 Axis Regulates the Invasiveness of Nasopharyngeal Carcinoma Cell Lines by Modulating EGFR Activation-Stimulated Switch from E- to N-Cadherin
Source: Molecules. 2023 Jan 13;28(2):818. doi: 10.3390/molecules28020818 (PMC9864293; doi:10.3390/molecules28020818)
Supplement: Supplementary file 1 [file molecules-28-00818-s001.zip › Supplementary Table S1.pdf]

Supplementary Table S1. Primer sequence and product length

Gene name		Primer sequence（5'→3'）	Product length
STIM1	Forward	CTCTCTTGACTCGCCATAATCA	101 bp
	Reverse	GTAACGGTTCTGGATATAGGCA	
GAPDH	Forward	GTGGACCTGACCTGCCGTCTAG	149 bp
	Reverse	GAGTGGGTGTCGCTGTTGAAGTC	
