# Supplementary figures and images for: The miRNA-185-5p/STIM1 Axis Regulates the Invasiveness of Nasopharyngeal Carcinoma Cell Lines by Modulating EGFR Activation-Stimulated Switch from E- to N-Cadherin
Source: Molecules. 2023 Jan 13;28(2):818. doi: 10.3390/molecules28020818 (PMC9864293; doi:10.3390/molecules28020818)

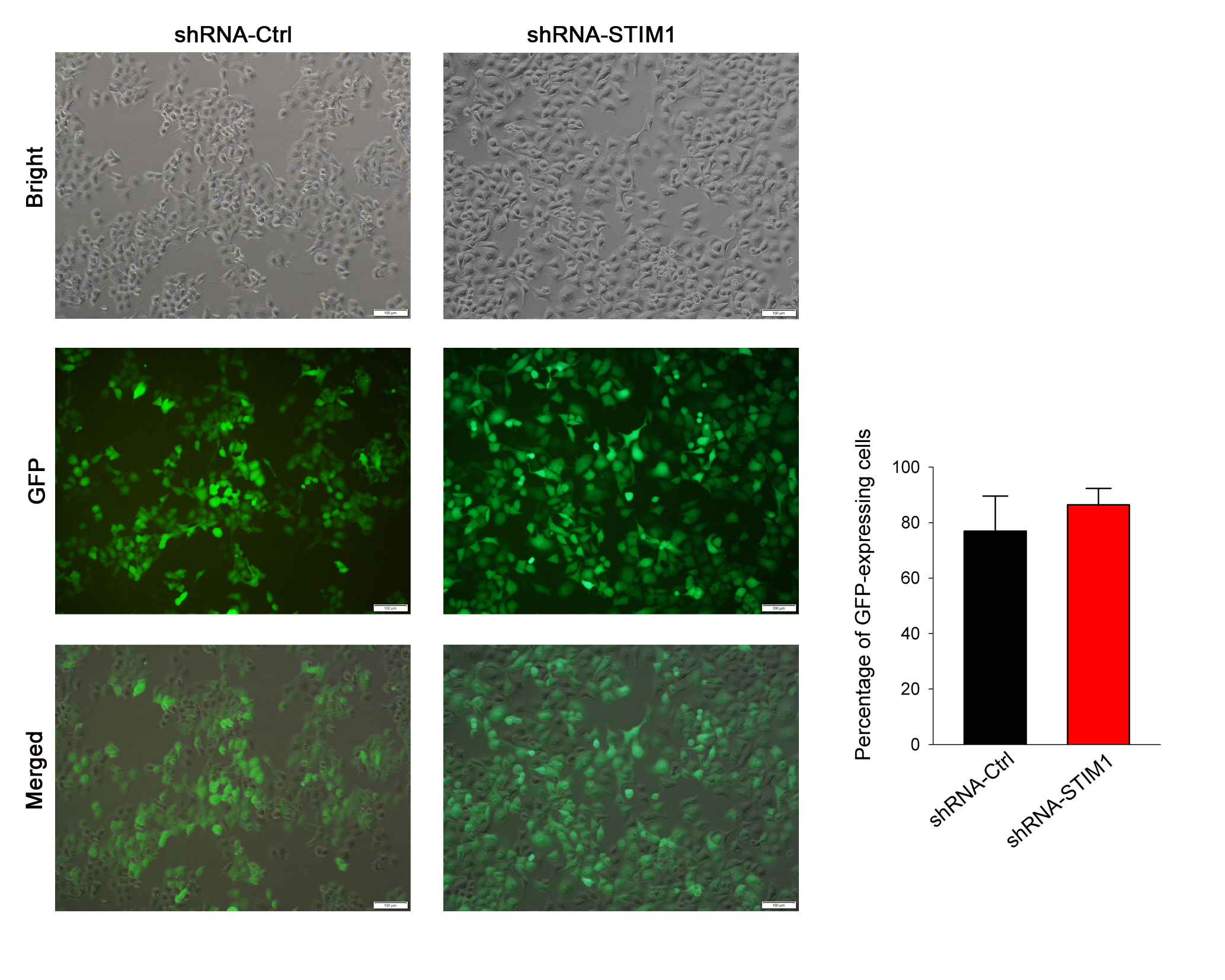

Supplement: Supplementary file 1 [file molecules-28-00818-s001.zip › Supplementary Figure S1.tif]

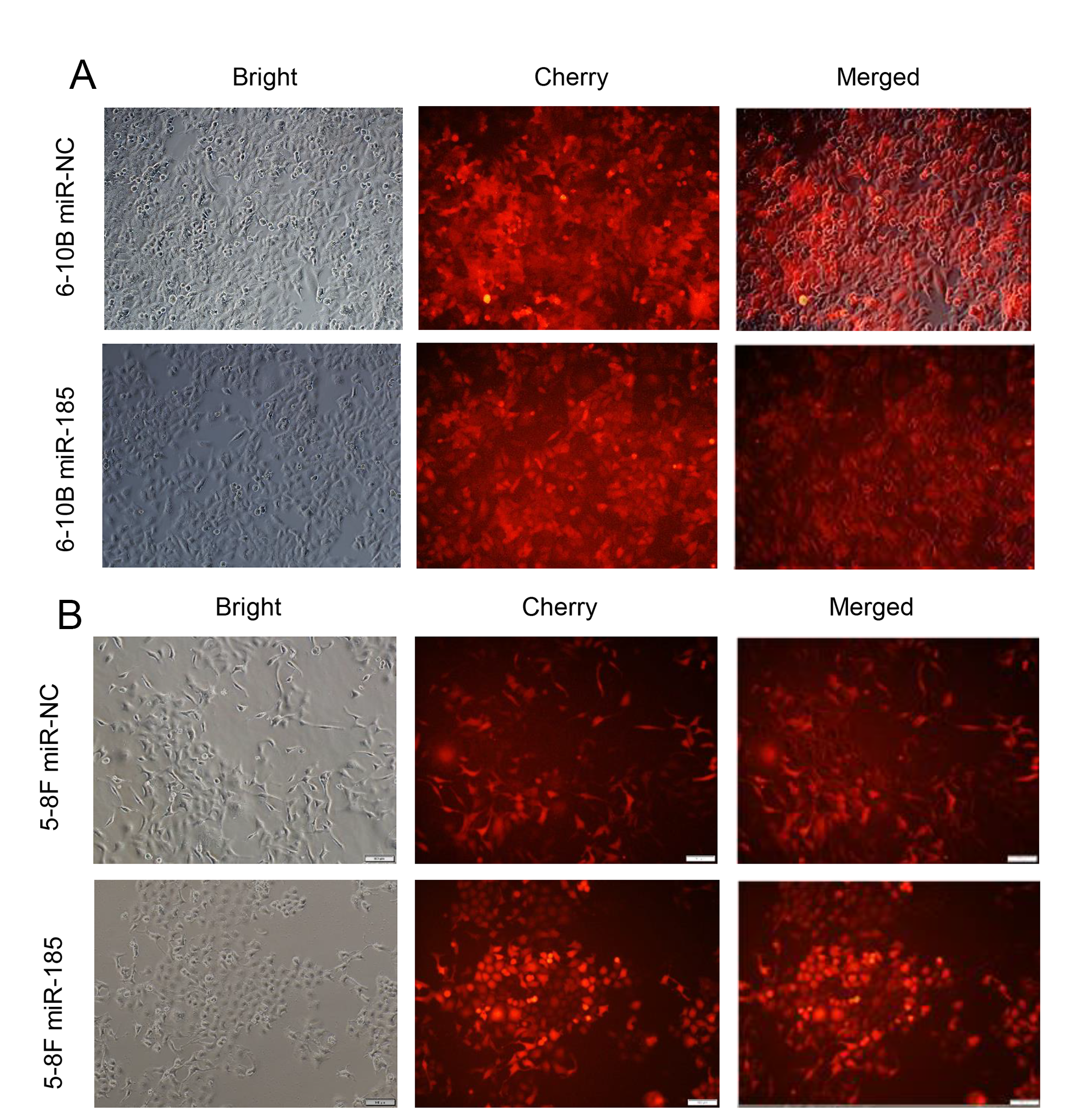

Supplement: Supplementary file 1 [file molecules-28-00818-s001.zip › Supplementary Figure S2.tif]
